# Serum Calcitonin-Negative Medullary Thyroid Carcinoma: A Case Series of 19 Patients in a Single Center

**DOI:** 10.3389/fendo.2021.747704

**Published:** 2021-11-05

**Authors:** Sun Jung Kim, Hyeok Jun Yun, Su-Jin Shin, Yong Sang Lee, Hang-Seok Chang

**Affiliations:** ^1^ Department of Surgery, Gangnam Severance Hospital, Yonsei University College of Medicine, Seoul, South Korea; ^2^ Department of Surgery, Thyroid Cancer Center, Gangnam Severance Hospital, Institute of Refractory Thyroid Cancer, Yonsei University College of Medicine, Seoul, South Korea; ^3^ Department of Pathology, Gangnam Severance Hospital, Yonsei University College of Medicine, Seoul, South Korea

**Keywords:** thyroid cancer, calcitonin, case series, medullary thyroid carcinoma (MTC), calcitonin-negative medullary thyroid carcinoma.

## Abstract

**Introduction:**

Medullary thyroid carcinoma (MTC) is a rare cancer that accounts for 5% of thyroid cancers. Serum calcitonin is a good biomarker for MTC, which is used for diagnosis, prognosis, and monitoring of recurrence. Calcitonin-negative MTC (CNMTC) is rare but confounds diagnostic and prognostic directions. This study introduces 19 cases of CNMTC in a single center.

**Method:**

From 2002 March to 2020 July, more than 76,500 patients had undergone thyroid surgery due to thyroid cancer at the Severance Hospital, and a total of 320 patients were diagnosed with MTC (0.4%). Serum calcitonin levels were obtained from every patient who was suspected with MTC. These patients had undergone either bilateral total thyroidectomy or unilateral thyroidectomy with central compartment lymph node dissection, and additional modified radical lymph node dissection if lateral lymph node metastasis was positive. Postoperative monitoring and out-patient clinic follow-up were performed with obtaining the serum calcitonin levels.

**Result:**

Nineteen patients tested negative for calcitonin preoperatively (6%). The mean preoperative calcitonin level was 5.1pg/mL if undetectable level is regarded as 0pg/mL. Only two patients were males, and the female bias was significant (p = 0.017). No one except two patients with modified radical neck dissection showed central compartment lymph node metastasis. Every patient’s postoperative calcitonin level remained low. The median follow-up period was 71 months. There was no recurrence and only one fatality, and the overall survival rate was 95%.

**Conclusion:**

Since incidence of CNMTC is not negligible, MTC should not be ruled out in the diagnostic phase even if serum calcitonin is negative in preoperative examination. We presented 19 cases of CNMTC whose prognosis in general were favorable. Markers of serum and immunohistochemical samples other than calcitonin should be actively examined.

## Introduction

Medullary thyroid carcinoma (MTC) is a neuroendocrine tumor originating from parafollicular C cells from the neural crest ectoderm and accounts for 5% of thyroid cancers (1, 2). The 10-year survival rate for MTC has increased from 50% to 60-80% (3). Serum calcitonin is a good indicator for MTC with high sensitivity and specificity. Elevated basal calcitonin and stimulated serum calcitonin with the pentagastrin test have good diagnostic values. Serum calcitonin also acts as a predictor for tumor prognosis and tumor recurrence. Meanwhile, there have been plenty of single case reports of MTC patients whose basal serum calcitonin level and stimulated calcitonin level were within normal range (upper normal level being 10 pg/ml and 1000 pg/ml, respectively), contrary to conventional values. Several case studies reported that immunohistochemical stains were also negative for calcitonin (4–8). Sobol et al. first reported such a case of MTC with normal serum calcitonin (8). Such tumors were called hormone-negative endocrine tumors, in that they did not secrete their resident hormones. Later, this type of tumor was termed atypical MTC because its immunohistochemical stain is negative for calcitonin but positive for neuroendocrine markers such as chromogranin A (CgA), synaptophysin, and neuron specific enolase (NSE) (9). Some called it non-secretory medullary thyroid cancer (NCR-MTC) because normal basal serum calcitonin observed in such cases seemed to be derived from defective secretory mechanism in these MTC (10). Today, such cases are commonly named calcitonin-negative medullary thyroid cancer (CNMTC) and the largest ever case series of CNMTC contained 19 patients (11). Our institutions have performed large amounts of surgical intervention for thyroid cancers including MTC. Hereby, we introduce 19 cases of CNMTC treated at the Severance Hospital across 19 years.

## Materials and Methods

From 2002 March to 2020 July, more than 76,500 patients had undergone thyroid surgery due to thyroid cancer at the Severance Hospital. Among them, 320 patients whose final pathological diagnosis was MTC (0.4%) were analyzed in a retrospective study. Preoperative thyroid sonography, neck computed tomography (CT) scan, and thyroid function test were done routinely. Pathologic confirmation was performed with sonography-guided fine needle biopsy. If suspected for advanced thyroid cancer, chest CT and positive emission tomography (PET)-CT were done to examine distal metastasis. Basal complete blood count, prothrombin times, activated partial thromboplastin time, chemistry labs such as levels of blood urea nitrogen/creatinine, electrolyte, liver enzymes, and chest radiograph were obtained. Serum calcitonin level was examined in all patients suspected for any kind of thyroid malignancy. Serum calcitonin level was measured by two different assays (Liaison XL and Immulite 2000) with the same principle of chemiluminescent immunometric assay. Every patient had undergone radical thyroidectomy with central compartment neck node dissection and additional modified radical neck node dissection if the lateral lymph node metastasis was present. Postoperative serum calcitonin was acquired on postoperative day 1. All patients were followed up regularly in outpatient clinic; 1~2 weeks after operation day, and every 6 months for 1~2 years, then yearly afterwards. Thyroid sonography with or without neck CT and serum calcitonin levels were obtained in the outpatient clinic.

For the study data analysis, categorical variables were described by frequency and proportion, while summary statistics (median, range) were used to report continuous data.

The Institutional Review Board of Gangnam Severance Hospital, Yonsei University College of Medicine (Seoul, Korea) approved this study, and the study protocol was conducted in accordance with the tenets of the Declaration of Helsinki. Due to the retrospective nature of the study, neither patient approval nor informed consent was required (approval number: 3-2020-0324).

## Results

A total of 320 patients diagnosed with MTC were examined. Male and female patients were 104 and 195 each (34.8% vs 65.2%). The mean and median ages were 49.4 and 50 years, respectively, with age ranging from 6 to 89 years. Basal calcitonin levels were obtained from all patients, and 301 patients (94%) had elevated basal calcitonin level, whereas 19 patients (6%) had normal preoperative serum calcitonin levels (< 10 pg/mL).

Among CNMTC patients, two patients were male (9.5%) while 17 patients were female (90.5%), and the female proportion was significantly higher than that observed in the secretory MTC population (p = 0.017) (**Table 1**). Mean and median ages were 50 and 49 years, respectively. Preoperative fine needle aspiration showed eight cases suspected for malignancy, one papillary thyroid cancer, and 10 MTC. All patients’ preoperative calcitonin level was obtained while only 13 patients’ preoperative carcinoembryonic antigen (CEA) level was obtained (**Table 2**). Calcitonin levels lower than 2 pg/mL were regarded as 0; hence the mean preoperative calcitonin level among CNMTC patients was 5.1 pg/mL. Procalcitonin level was checked in one patient, which was within the normal range. One patient was clinically suspected to have multiple lung metastasis.

**Table 1:** Clinical characteristics of calcitonin-negative medullary thyroid carcinoma patients.

Variable	n = 19
Age (year), mean ± SD	50.1 ± 11.8
Sex (F/M), n (%)	17/2 (89.5/10.5)
Tumor diameter (cm), mean ± SD	0.68 ± 0.55
Tumor multiplicity, n (%)	3 (15.8)
Tumor capsule invasion, n (%)	3 (15.8)
Thyroidectomy (less than total/total), n (%)	6/13 (31.6/68.4)
Lymphadenectomy (CCND/MRND), n (%)	19/2 (100/10.5)
Retrieved LNs, mean (range)	7.9 (0–46)
Metastatic LNs, mean (range)	1.3 (0-23)
T stage, n (%)	
T1	16 (84.2)
T2	1 (5.3)
T3	1 (5.3)
T4a	1 (5.3)
N stage, n (%)	
N0	17 (89.5)
N1b	2 (10.5)
M stage, n (%)	
M0	18 (94.7)
M1	1 (5.3)
Stage, n (%)	
Stage I	16 (84.2)
Stage II	2 (10.5)
Stage III	1 (5.3)
Follow up duration (month), median (range)	71 (4-162)

SD, Standard deviation; CCND, central compartment neck node dissection; MRND, modified radical neck node dissection; LN, lymph node.

**Table 2:** Perioperative serum calcitonin and CEA level and histopathologic findings of calcitonin-negative medullary thyroid carcinoma.

Patient No.	Sex	Age (years)	Preop-CT (pg/mL)	Preop-CEA (ng/mL)	Immediate Postop-CT (pg/mL)	Postop-CEA (ng/mL)	IHC-CT	IHC-CEA	IHC-CgA	RET^a^
**1**	F	80	8.1	2.0	1.7	1.7	–	NA	+	–
**2**	F	31	2.6	NA	NA	1.2	+	+	+	–
**3**	F	49	3.2	NA	4.3	12.6	+	+	NA	NA
**4**	F	41	8.2	3.4	NA	2.6	+	+	NA	NA
**5**	F	52	6.0	NA	1.5	1.3	NA	NA	NA	NA
**6**	F	60	7.2	0.5	NA	0.5	+	+	NA	NA
**7**	F	52	3.6	2.2	2.6	1.0	+	+	+	+
**8**	F	75	2.0	1.1	NA	1.2	–	–	NA	NA
**9**	F	53	5.3	0.9	NA	0.7	–	NA	NA	+
**10**	F	47	3.0	1.2	NA	0.9	+	+	NA	+
**11**	F	44	<2	NA	<2	2.9	+	+	NA	–
**12**	M	49	7.0	NA	<2	7.6	+	+	NA	NA
**13**	F	45	<2	NA	21.6	0.6	–	–	–	+
**14**	F	45	3.2	1.2	<2	0.6	+	+	NA	NA
**15**	M	39	5.2	2.0	<2	1.6	+	NA	NA	NA
**16**	F	49	4.7	1.3	1.0	1.7	+	–	NA	–
**17**	F	57	4.3	7.1	<1	7.2	+	NA	+	NA
**18**	F	46	8.1	4.4	NA	2.5	+	NA	+	–
**19**	F	38	<2	1.0	<2	0.4				

CT, calcitonin; CEA, carcinoembryonic antigen; CgA, chromogranin A; IHC, immunohistochemistry; NA, not available.

a RET mutation.

There were 16 patients out of 19 who had single lesions, while three patients showed multifocal lesions; one was restricted to the ipsilateral lobe and the other two had bilateral cancers. There were 11 patients out of 19 who underwent bilateral total thyroidectomy with central compartment lymph node dissection, while 6 patients underwent unilateral thyroidectomy with central compartment lymph node dissection. The other two patients underwent modified radical neck dissection (MRND) with bilateral total thyroidectomy, with central compartment lymph node dissection. The mean number of retrieved lymph nodes were 7.9, ranging from 0 to 46 (**Table 1**).

**Figure 1:**
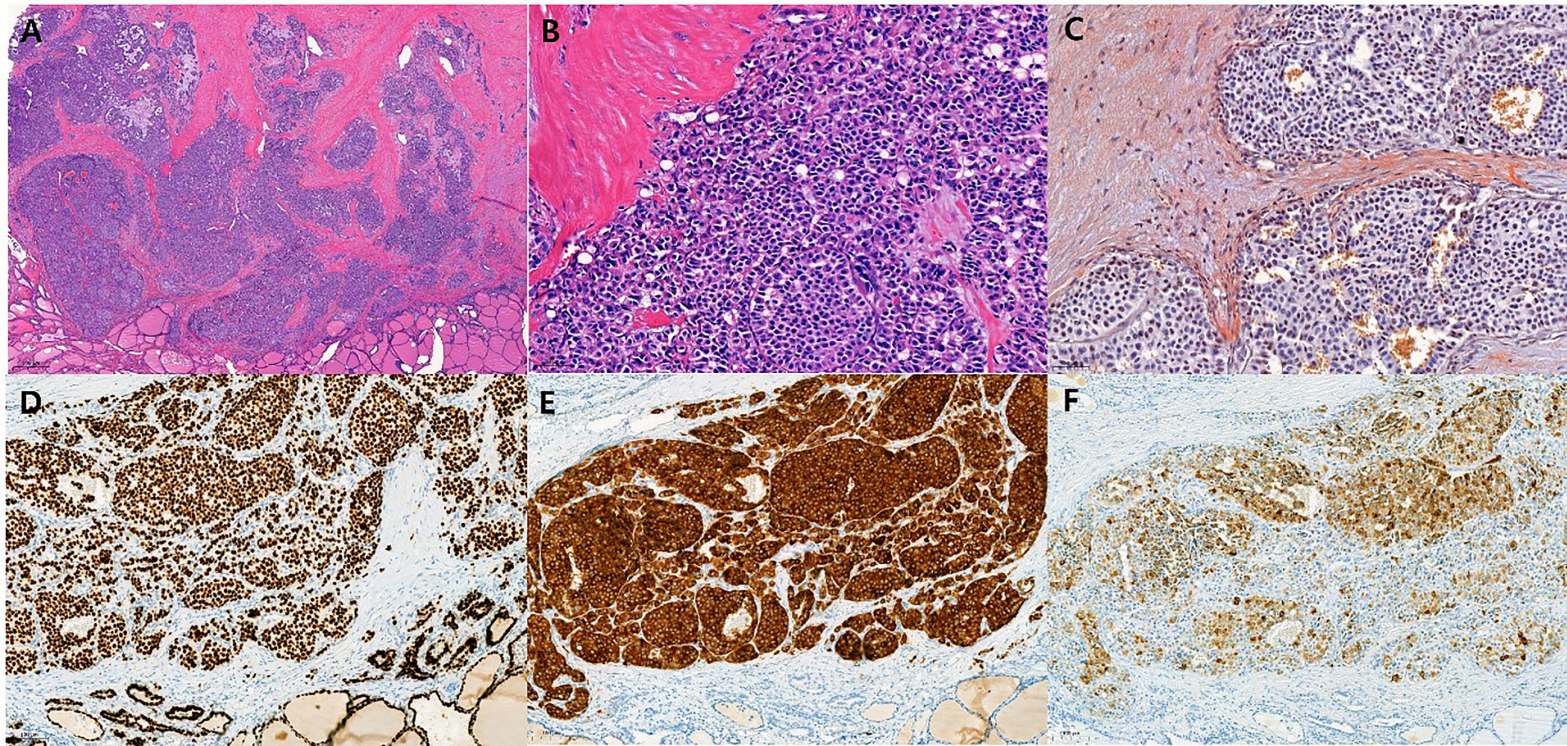
Histopathologic findings of calcitonin-negative medullary thyroid carcinoma **(A)** The tumor shows solid sheets and nests growth patterns with fibrotic stroma. (H&E, ×20) **(B)** The polygonal tumor cells contain round to oval nuclei with coarse chromatin. Nucleoli are not prominent and the cytoplasm is granular and eosinophilic. (H&E, ×200) **(C)** The amyloid deposition in stroma is recognized by Congo red stain with polarized microscopic examination (Congo red, ×200). The tumor cells show strong and diffuse immunoreactivity for TTF-1 [**(D)**, ×100] and synaptophysin [**(E)**, ×100], and show weak to strong heterogeneous immunoreactivity for calcitonin [**(F)**, ×100].

Final tumor pathological stage showed 16 T1, and one each of T2, T3, and T4. The mean tumor size was 6.8 mm. All except two MRND cases showed central compartment lymph node metastasis (**Table 1**). Only in the MRND cases were lymph node metastasis and capsule invasion seen; surprisingly 23 lymph node metastasis were found in a bilateral MRND case, who was preoperatively suspected to have lung metastasis, which was confirmed postoperatively. Immunohistochemical stain (IHC) showed that 14 patients were calcitonin-positive, and nine patients were CEA-positive. Other stains such as CgA, synaptophysin, CK, and CD56 were positive in 5, 1, 1, and 3 patients, respectively. Among 14 patients with positive calcitonin stain, CEA was positive in nine patients and negative in one patient who had undergone IHC stain for CEA. There were four patients who underwent CgA stain, and they were all positive for calcitonin and CgA (**Table 2**). The gross microscopic finding of CNMTC was no different to that of the typical MTC. The tumor consists of amyloid-deposited fibrous stroma and polygonal tumor cells with strong immunoreactivity for thyroid transcription factor-1 and synaptophysin (**Figure 1**).

**Figure 2:**
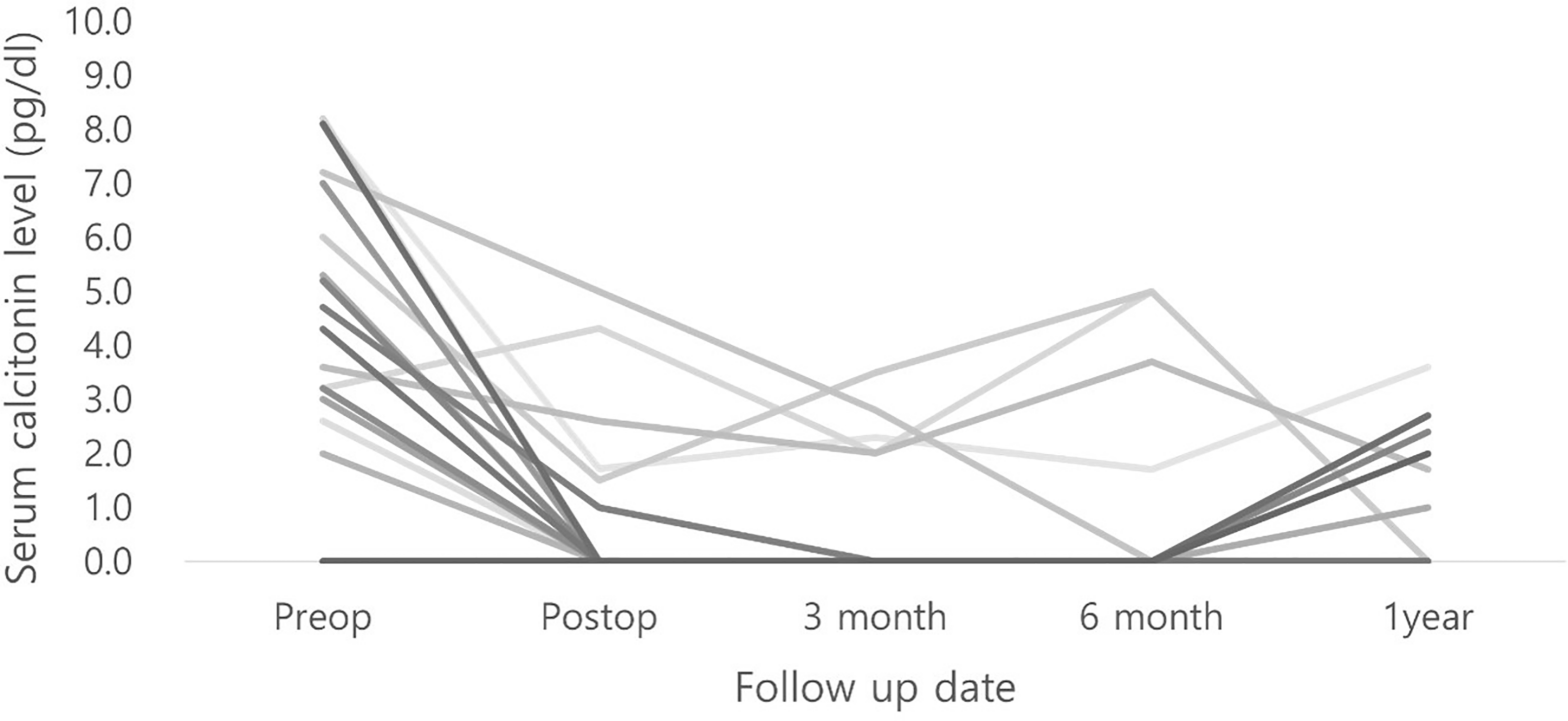
Serum calcitonin levels of calcitonin-negative medullary thyroid carcinoma patients.

Immediate postoperative calcitonin levels and those 2 or 3 days postoperatively were obtained. The mean immediate postoperative calcitonin level was 5.5 pg/dL; however, the immediate serum calcitonin levels of 8 patients were not available. Serum calcitonin levels were followed up for several months across intervals in outpatient clinics, and they had not increased beyond normal levels in all other patients (**Figure 2**).

All patients were followed up thoroughly in outpatient clinics. The median follow-up period was 71 months. One case of mortality was observed in a patient who was initially suspected with lung metastasis, which was confirmed after surgical resection and follow-up re-examination. She underwent wedge resection of the lung under video-assisted thoracoscopic surgery 3 months after thyroid operation. Despite subsequent palliative chemotherapy, the patient developed brain metastasis 5 years after the initial diagnosis and died 1 month later. Other than this case, no recurrences were reported. The overall survival was 94.7%.

## Discussion

MTC is a neuroendocrine tumor originating from parafollicular C cells (1). First described by Hazard in 1959, parafollicular cells are derived from the neural crest ectoderm and ultimobranchial body and account for 1% of the thyroid cells (12). Parafollicular C cells are responsible for calcium homeostasis by synthesizing and secreting calcitonin. MTC accounts for 5% of thyroid cancers and has a mean survival rate of 8.6 years, with the 10-year survival rate ranging from 69% to 89% (3, 13). It mostly involves lymph node metastasis and involves distant metastasis in 20% of cases (14). Lymph node metastasis tends to increase according the size of the MTC (11). MTC occurs sporadically in 75% of cases and is hereditary in 25% of cases; the latter being associated with germline mutation of the RET proto-oncogene on chromosome 10 (15). This familial form generally presents in combination with other neuroendocrine diseases, termed multiple endocrine neoplasia (MEN). MEN2A occurs with pheochromocytoma and hyperparathyroidism, and MEN2B, common in childhood, occurs with pheochromocytoma, mucosal neuromas, gastrointestinal neurogangliomatosis, and megacolon. Sporadic form of MTC is usually a well-differentiated, slow growing, and locally aggressive tumor. Meanwhile, familial MTC has worse prognosis with more involvement of lymph node metastasis, adjacent organ invasion, and hematogenous dissemination (13).

Medullary tumor cell is a round cell that distinctively produces amyloid substance and has separated fibrous stroma (12). Medullary thyroid cancer characteristically synthesizes and secretes calcitonin, which is used for early diagnosis and identification of MTC and also postoperative monitoring (16). Serum calcitonin is usually associated to tumor size and volume, and general tumor burden (17, 18). Basal serum calcitonin level above 100 pg/ml shows high sensitivity and specificity of the disease. If needed, the pentagastrin stimulating test is done, where a stimulated serum calcitonin level above 1000 pg/ml suggests the possibility of MTC. Nowadays, diagnostic tools such as fine-needle cytology (FNC) washout fluids and serum procalcitonin or calcium stimulation of calcitonin have been proposed (19–21). Calcitonin can be falsely elevated or decreased in C cell hyperplasia, autoimmune thyroiditis, end stage renal disease, lung and prostate cancer, and some neuroendocrine tumors (22). Sometimes, in millimetric MTC, serum calcitonin levels can be in the normal range, but this rarely is the case in voluminous and palpable MTC (13). Serum calcitonin is also associated with recurrence, as persistent postoperative elevation of serum calcitonin is related to high rates of local and distant metastasis and finally mortality (23).

Medullary thyroid carcinoma with normal or low serum calcitonin was first reported in 1989 (8). It was referred to using several different names such as atypical MTC, non-secretory MTC, and finally CNMTC (8–11). Up to date, there are more than 50 single case reports of CNMTC, and the largest case series of CNMTC was done with 19 patients (11). So far, several reports have been published with few numbers of CNMTC cases. In large volume studies regarding medullary thyroid cancer, the incidence of CNMTC was only about 1% (10, 24). The prevalence of CNMTC in this study was 6% (19 out of 320).

Serum markers other than calcitonin can be used for the diagnosis of CNMTC for the following reasons. MTC also synthesizes and secretes molecules such as CEA, CgA, synaptophysin, NSE, and CK7, which can sometimes be useful serum markers for diagnosis and follow-up (25). Serum CEA and CgA levels can be elevated in MTC when performing pentagastrin stimulating test (1, 6, 26). IHC staining of CEA and chromogranin can be used in the diagnosis in addition to calcitonin. As CEA is also synthesized and secreted in the same cell population, serum and IHC CEA might be a good adjunct biomarker (27). Procalcitonin is a polypeptide calcitonin precursor that is stable with a half-life of 24 hrs. It is comparable to calcitonin in identifying primary MTC and/or local/distant advancements (9). Calcitonin gene-related peptide (CGRP) is a neuropeptide secreted from MTC cells and non-neoplastic C cells rather than C cell hyperplasia, and occurs from the alternative RNA splicing of CALC-1 gene (22). CGRP is said to be used in distinguishing non-secretory MTC and thyroid neuroendocrine tumor. In this study, the majority of patients had sampled serum CEA, and only one had slightly elevated CEA. Preoperative serum procalcitonin was performed in only one patient, and none of the cases had preoperative CGRP measures. The importance of CgA in the identification of CNMTC is emphasized by following reasons. The possibility of chromogranionoma of MTC was first suggested due to elevated serum CgA level (8). Also, a case series reported that almost all cases showed strong positivity of CgA, in spite of weak or focal IHC stain of calcitonin (10). In the present study, chromogranin A stain was performed only in six patients, but five of them showed positive stains. We cautiously suggest that if the other patients had undergone CgA statins, a majority of them could have shown positive stains for CgA.

Meanwhile differentiating MTC from neuroendocrine tumor (26) can be troublesome due to their morphological and immunohistochemical similarities. Both cells are spindle-shaped or round cell in trabecular arrangement with amyloid deposits. Also they commonly stain positive for CgA, NSE, and CEA. Thus it is important to confirm IHC staining of calcitonin (5). In this study, 15 out of the 19 cases stained positive for calcitonin in IHC and nine out of 12 cases that had underwent CEA stains were positive for CEA. Positive IHC stains for calcitonin tended to relate with positive IHC stain for CEA (p = 0.045). Furthermore, four out of five CgA-positive stain cases showed calcitonin positivity, whereas one CgA negative stain case was negative for the calcitonin stain. More data on IHC staining would have been appropriate to assist differential diagnosis of CNMTC.

The pathophysiology of CNMTC is not well known. Some say it is calcitonin assay interference and others say it is the pathological status where malignant parafollicular cells lose control of their synthesizing or secreting functions (1, 3, 22). The proportion of calcitonin or its precursor secreted by neoplastic C cells can differ in CNMTC (10, 28). Aggressive and undifferentiated MTC losses the ability to secrete calcitonin, and its prognosis is worse (29). In our study, we persistently used the same calcitonin assay method. The serum calcitonin level of the 19 patients in our study constantly stayed low. The possibility of the hook effect in the calcitonin assay seemed low.

Treatment of CNMTC is not different to that of MTC. Total thyroidectomy with adequate lymphadenectomy is necessary. Variation in the extent of surgery in our study was due to tumor multiplicity, capsule invasion, and suspicious findings of lymph node metastasis. Preoperative thyroid sonography, CT scan, magnetic resonance imaging scan, as well as serum thyroid function tests are needed. Serum calcitonin is used to detect the possibility of MTC, but the disease activity of MTC might not relate to serum calcitonin levels (4). As our case series shows that 6% of MTC have non-elevated serum calcitonin, preoperative diagnosis should be made with sufficient examination of serum biomarkers such as CEA, procalcitonin, and CGRP. Further, examinations of calcitonin mRNA and DNA *via in situ* hybridization might be useful when serum calcitonin level is normal and where morphologically MTC is suspected (4).

The oncological outcome of CNMTC depends on serum calcitonin level (11). In general, considering that the deceased expired patient already had distant metastasis at the time of diagnosis, the prognosis of CNMTC in the 19 patients in our study was favorable with only one death case with distant metastasis. Meanwhile some suggest that the prognosis of CNMTC patients differed according to Ki67 expression or RET mutation (10). In this study, 10 patients out of 19 underwent RET mutation analysis, where four patients had mutations. Three patients had single chromosome mutation whereas one patient had three different RET mutations concurrently. There was no recurrent case and there was one expired case, with 5-year survival rate 94.7%. Comparing prognosis according to RET mutations was not possible due to the limited examination of RET mutations in our cohort.

The limitation of this study lies in its precondition upon the case series. Only observational analysis was possible due to the small number of subjects. Also, the dates when the serum calcitonin levels were obtained were not regular, and acquisition of other serum markers was scarce. IHC stains other than calcitonin were irregularly performed. However, this is probably because biomarkers other than calcitonin were not standardized for serum sampling and immunohistochemical staining.

## Conclusion

Since the incidence of CNMTC is not negligible, MTC should not be ruled out in the diagnostic stage even if serum calcitonin is negative on preoperative examination. We presented 19 cases of CNMTC with generally favorable prognosis. Markers for serum sampling and IHC stain other than calcitonin should be actively performed after obtaining negative calcitonin results.

## Data Availability Statement

The original contributions presented in the study are included in the article/supplementary material. Further inquiries can be directed to the corresponding author.

## Ethics Statement

The studies involving human participants were reviewed and approved by The Institutional Review Board of Gangnam Severance Hospital, Yonsei University College of Medicine (Seoul, Korea). Written informed consent for participation was not required for this study in accordance with the national legislation and the institutional requirements.

## Author Contributions

Study concept and design: YL. Acquisition, analysis, or interpretation of data: SK, HY, and S-JS. Drafting of the manuscript: SK, HY, YL, and H-SC. Statistical analysis: SK and HY. All authors contributed to the article and approved the submitted version.

## Funding

This research was supported by Basic Science Research Program through the National Research Foundation of Korea (NRF) funded by the Ministry of Science and ICT (2017R1E1A1A03070345).

## Conflict of Interest

The authors declare that the research was conducted in the absence of any commercial or financial relationships that could be construed as a potential conflict of interest.

## Publisher’s Note

All claims expressed in this article are solely those of the authors and do not necessarily represent those of their affiliated organizations, or those of the publisher, the editors and the reviewers. Any product that may be evaluated in this article, or claim that may be made by its manufacturer, is not guaranteed or endorsed by the publisher.
